# Time to First Line Antiretroviral Therapy Adverse Drug Reaction and its Predictors Among Adult HIV/AIDS Patients on Treatment in Eastern Ethiopia

**DOI:** 10.3389/fphar.2022.922744

**Published:** 2022-08-15

**Authors:** Adisu Birhanu Weldesenbet, Biruk Shalmeno Tusa, Gebiso Roba Debele, Malede Mequanent Sisay, Tadesse Awoke Ayele

**Affiliations:** ^1^ Department of Epidemiology and Biostatistics, School of Public Health, College of Health and Medical Sciences, Haramaya University, Haramaya, Ethiopia; ^2^ Department of Public Health, College of Health Sciences, Mettu University, Mettu, Ethiopia; ^3^ Department of Epidemiology and Biostatistics, Institute of Public Health, College of Medicine and Health Sciences, University of Gondar, Gondar, Ethiopia

**Keywords:** ART, ADR, hiv/aids, predictors, adults, Ethiopia

## Abstract

**Background:** Even though determining the time to anti-retroviral therapy (ART) adverse drug reaction and its predictors is a crucial step to overcome the negative consequences of the adverse drug reaction, there is limited information regarding the time to ART adverse drug reaction and its predictors. Therefore, this study aimed to determine the time to first ART adverse drug reaction and its predictors among adult HIV/AIDS patients on first-line antiretroviral therapy in West Hararghe Zone, Eastern Ethiopia.

**Methods:** An institution-based retrospective cohort study was conducted on 561 HIV/AIDS patients on first-line ART from September 2013–January 2019 at public hospitals in West Hararghe Zone, Eastern Ethiopia. Data were collected using checklists and document reviews, entered using Epi Info and analyzed in R software. A Cox proportional hazard model was fitted to identify predictors of the time to first ART adverse drug reaction. Model adequacy was checked using Cox Snell residuals. An adjusted hazard ratio with its confidence interval was used to show the presence and strength of association at a 95% confidence level.

**Result:** Most (90.74%) ART adverse drug reactions occurred within 1 year of initiation of ART. Overall, 54 patients developed ART adverse drug reactions with an incidence density of 3.5/100 persons-years of observations (95% CI: 2.7–4.6). The initial ART regimen (TDF, 3TC, EFV) [AHR = 0.3, 95% CI 0.1–0.7], fair adherence [AHR = 8.8, 95% CI 3.3–23.2], poor adherence [AHR = 7.8, 95% CI 3.1–19.5], moderate body mass index (BMI) at the baseline [AHR = 4.4, 95% CI 1.8–11.0], severe body mass index [AHR = 2.8, 95% CI 1.1–6.8], World Health Organization (WHO) stage II [AHR = 3.7, 95% CI 1.2–11.3] and WHO stage IV [AHR = 6.3, 95% CI 2.0–19.8] were significant predictors of the time to ART adverse drug reactions.

**Conclusion:** In conclusion, most of the ART adverse drug reactions occurred within 1 year of initiation of ART. The initial ART regimen (TDF, 3TC, EFV), adherence, HIV/AIDS stage, and BMI were risk factors for the time to ART adverse drug reaction. The incidence of the antiretroviral therapy adverse reaction was relatively low with early onset. Close monitoring of clients in clinical stage II and above is required and continuous assessment for improving the detection and management of adverse drug reactions is recommended. Patients with poor adherence need to get continuous counseling to improve their adherence status.

## Introduction

Globally, 36.9 million people are living with human immunodeficiency virus (HIV) and more than half of them are in Africa (WHO, 2019). At the end of 2017, 21.7 million (59%) patients were receiving antiretroviral therapy (ART). Consequently, acquired immunodeficiency syndrome (AIDS)-related mortality was reduced by around 51% globally (WHO, 2017). In Ethiopia, reports indicate that the overall prevalence of HIV/AIDS is 1.1% (Girum et al., 2018). Timely initiation of ART and appropriate monitoring of treatment effectiveness improve the life expectancy of people living with HIV/AIDS (Daniel Murrell, 2018).

Antiretroviral therapy-related adverse drug reactions are one of the challenges to the United Nations’ target set to end AIDS epidemics by 2030 (WHO, 2013)**.** Antiretroviral therapy adverse drug reactions may cause discontinuation of all antiretroviral drugs and make HIV/AIDS patients susceptible to life-threatening chronic diseases (Carr and Cooper, 2000; Hicks et al., 2006).

Globally, the incidence of adverse drug reactions among patients on first-line ART ranges between 11 and 35.9% (Hogg et al., 1999; Bonfanti et al., 2000; Severe et al., 2010). In Ethiopia, a study from Debre Markos reported that 51.5% of patients on first-line ART experienced ART adverse drug reactions (Asrat et al., 2014). Furthermore, ART adverse drug reactions are common reasons for poor adherence to antiretroviral drugs (Duval et al., 2004; Masenyetse et al., 2015; Kindie et al., 2017).

To improve the survival of HIV/AIDS patients on ART, information on factors associated with the time to ART adverse drug reactions and its predictors are needed. However, there is a paucity of evidence on the time to ART adverse drug reactions and its predictors among HIV/AIDS patients in Ethiopia. Although few studies were conducted on the time to ART adverse drug reactions, they did not assess the effect of the initial ART regimen, comorbidity, and adherence on the time to ART adverse drug reactions (Kindie et al., 2017; Asfaw and Enquselassie, 2018a). Therefore, the present study aimed to determine the time to ART adverse drug reactions and its predictors among HIV/AIDS patients at public hospitals in West Hararghe Zone, Eastern Ethiopia, from 2013–2019. The finding of the present study will be helpful for the timely identification and treatment of ART adverse drug reactions and to decrease the progression of adverse drug reactions and related complications.

## Methods

### Study Design and Settings

An institution-based retrospective cohort study was conducted among adult HIV/AIDS patients on ART from September 2013–January 2019 at public hospitals in West Hararghe Zone of the Oromia region, Eastern Ethiopia. Among five hospitals in the zone, the study was conducted in Chiro Zonal Hospital and Gelemso General Hospital because of the availability of ART data. The two hospitals render services to around 2,300,000 people in the zone. In these hospitals, 1,711 patients were on ART at the time of the survey.

### Study Population

The study included all HIV/AIDS patients aged 18 years and more who started ART between 2013 and 2019 and registered in the ART registry log book from Chiro and Gelemso Hospitals. Patients whose date of treatment initiation was not recorded, transferred in from other treatment centers, patients who have been receiving ART for less than 3 months, and pregnant mothers were excluded from the study.

### Sample Size Determination

The sample size was determined by STATA software after considering Cox proportional hazard model assumptions. We assumed a probability of type I error (*α*) 0.05, power of the study of 80%, and withdrawal probability of 0.1 which are the proportions of the subjects anticipated to withdraw from the study.

By taking the hazard ratio (HR) for strong predictors of adverse drug reactions (tuberculosis (TB) co-infection (HR = 2.5), World Health Organization (WHO) stage (HR = 2.2), and opportunistic infection prophylaxis (OIP) (HR = 3.2)) from a study in Bahir Dar, Ethiopia (Kindie et al., 2017), the calculated sample size is 412, 556 and 256, respectively. Therefore, 556 was taken as a minimum adequate sample size. Among 1,711 patients on ART at the time of the survey, 561 HIV/AIDS patients met the inclusion criteria and all of them were included in the study.

### Operational Definitions

**Antiretroviral therapy (ATR) adverse drug reaction (ADR):** The patient is said to have experienced ART adverse drug reactions if either hospitalization or discontinuation of treatment and switching of drugs to other ART regimen happened as a result of an undesired and excessive response to ART (Pharmacists ASoH-, 1995; Organization, 2008; Kindie et al., 2017). In the present study, at both hospitals, physicians’ diagnoses on patient cards and laboratory reports were used to check whether the patient developed ART adverse drug reactions.

In this study, baseline adherence (when the patient is first started on ART until the initial 30/60 dose was taken) was considered and accordingly classified in the following ways:

**Good adherence:** In the present study, if it is ≥ 95% or (<2 doses of 30 doses or <30 doses of 60 doses is missed) as documented by the ART healthcare provider (Nabukeera-Barungi et al., 2007; Chalker et al., 2009).

**Fair adherence:** The patient who missed 3–5 doses of 30 doses or 3–9 doses of 60 doses as documented by the ART healthcare provider is said to have fair adherence (Nabukeera-Barungi et al., 2007; Chalker et al., 2009).

**Poor adherence:** The patient who missed >6 doses of 30 doses or >9 doses of 60 doses as documented by the ART healthcare provider is said to have poor adherence (Nabukeera-Barungi et al., 2007; Chalker et al., 2009).

**Time to ART adverse drug reaction:** The time gap in days between being put on ART to the development of the first episode of ART adverse drug reactions.

**Censored:** Patients who did not experience ART adverse drug reactions during the follow-up period, died before experiencing ART adverse drug reactions within the study period, or discontinued ART before experiencing the event within the study period by reason not related to the event were counted as censored.

### Measurement of Variables

Data for this study were collected from patients’ routine records from the two hospitals. Data were extracted by reviewing the follow-up charts, ART registration log books, and patients’ intake forms. The health management information system card number was used to identify individual patient cards. The dependent variable for this study was the time to ART adverse drug reactions. The event of interest is the occurrence of the adverse drug reaction within the follow-up period. In the present study, the minimum follow-up period was 1 month. Patients were followed-up for a total of 5 years and 5 months.

Socio-demographic variables (age, sex, educational status, occupational status, place of residence, and marital status), baseline clinical variables (WHO HIV/AIDS stage, baseline CD4 count, body mass index (BMI), initial ART regimen, TB co-infection, OIP, baseline functional status, and adherence), and comorbidity were independent variables.

### Data Quality Assurance

By conducting a preliminary review, the adequacy of the checklist was evaluated and variables which are unavailable on records were excluded from the checklist. Trained healthcare workers who are familiar with chronic diseases were assigned as data collectors. In addition, to ensure data quality, a filled checklist was checked for consistency and completeness. Strict supervision was applied by supervisors during data collection.

### Data Processing and Analysis

The data were entered into Epi Info 7 and then exported to R statistical software 3.4.4 for analysis. Descriptive measures such as means and standard deviations were used to characterize the study population for quantitative variables whereas percentage and frequencies were used for categorical variables. The time to ART adverse drug reactions was estimated using the Kaplan–Meier (KM) method and the log-rank test was used to compare the survival time between groups of categorical variables. Proportional hazards assumption (PHA) was checked before fitting the Cox proportional hazard model by a cumulative log hazard plot. The factors significantly associated with ART adverse drug reactions in the bi-variable analysis at *p-*values less than 0.2 were included in the multivariable Cox regression model. The adjusted hazard ratio (AHR) with its confidence interval was used to show the presence and strength of association at a 95% confidence level. Overall adequacy of the model was checked using Cox Snell residuals.

## Results

### Baseline Description

A total of 561 HIV/AIDS patients were included in this study. The baseline age of the participants ranged from 18–64 years with a mean age of 33.4 (±8.9) years. Of the participants, 356 (63.5%) were females, 351 (62.6%) were urban dwellers, 139 (24.8) were non-government employed, and 304 (54.2%) were married (Table 1).

**TABLE 1:** Baseline socio-demographic characteristics of HIV/AIDS patients on ART in Eastern Ethiopia, 2013–2019.

Variables	Category	Frequency (*n* = 561)	Percent (%)
Sex	Male	205	36.5
	Female	356	63.5
Age	Mean (SD)	33.4 ± 8.9	
Marital status	Single	71	12.7
	Married	304	54.2
	Divorced	87	15.5
	Widowed	63	11.2
	Separated	36	6.4
Occupational status	Government employed	58	10.3
	Non-government employed	139	24.8
	Farmer	107	19.1
	Student	43	7.7
	Housewife	119	21.2
	Merchant	68	12.1
	Others	27	4.8
Place of residence	Urban	351	62.6
	Rural	210	37.4
Educational status	No formal education	167	29.8
	Primary	205	36.5
	Secondary	112	19.9
	Tertiary and above	77	13.7

Others = (Daily laborer, Driver), SD: standard deviation.

Of the total participants, 158 (28.2%) and 26 (4.6%) were in WHO stage III and IV, respectively, at the baseline, 100 (17.8%) had TB co-infection, 494 (88.1%) received OIP, and 358 (63.8%) had a baseline CD4 count of more than 200. Poor adherence was reported by 48 (8.6) HIV/AIDS patients. The most commonly prescribed initial ART regimen is TDF, 3 TC, and EFV. The most common types of ART adverse drug reactions encountered were anemia (33.9%) and rash (26.4%) (Table 2).

**TABLE 2:** Clinical characteristics of HIV/AIDS patients on ART in Eastern Ethiopia, from 2013–2019.

Variables	Category	Frequency (*n* = 561)	Percent (%)
WHO retroviral infection stage	Stage 1	285	50.8
	Stage 2	92	16.4
	Stage 3	158	28.2
	Stage 4	26	4.6
BMI	Normal	410	73.1
	Moderate	82	14.6
	Severe	69	12.3
Functional status	Working	470	84.8
	Ambulatory	72	12.8
	Bedridden	19	3.4
TB co-infection	No	461	82.2
	Yes	100	17.8
Adherence	Good	482	86.9
	Fair	31	5.5
	Poor	48	8.6
OIP	No	67	11.9
	Yes	494	88.1
CD4 count	≤200	203	36.2
	>200	358	63.8
Comorbidity	No	528	94.1
	Yes	33	5.9
ART regimen	AZT, 3TC, NVP	30	5.3
	AZT, 3TC, EFV	24	4.3
	TDF, 3TC, NVP	18	3.2
	TDF, 3TC, EFV	489	86.9
Type of ART adverse drug reaction	Anemia	18	33.3
	Diarrhea	14	25.9
	Rash	10	18.5
	Pain (tingling of extremities)	9	16.7
	Other	3	5.6

BMI: body mass index; TB: tuberculosis; OIP: opportunistic infection prophylaxis; AZT: zidovudine; 3 TC: lamivudine; NVP: nevirapine; EFV: efavirenz; TDF: tenefovir.

### Median Survival Time

The median survival time was not reached which means the study ended before 50% of the participants experienced ART adverse drug reactions.

### Survival Probability and Timing of ART Adverse Drug Reaction

The cumulative probability of survival without developing an adverse drug reaction at the end of one and a half years was 0.91 and 0.90 years, respectively, and the cumulative probability of survival by the end of the follow-up period was found to be 0.89. Regarding the timing of ART adverse drug reactions, most (90.7%) ART adverse drug reactions occurred within 1 year of initiation of ART.

**FIGURE 1:**
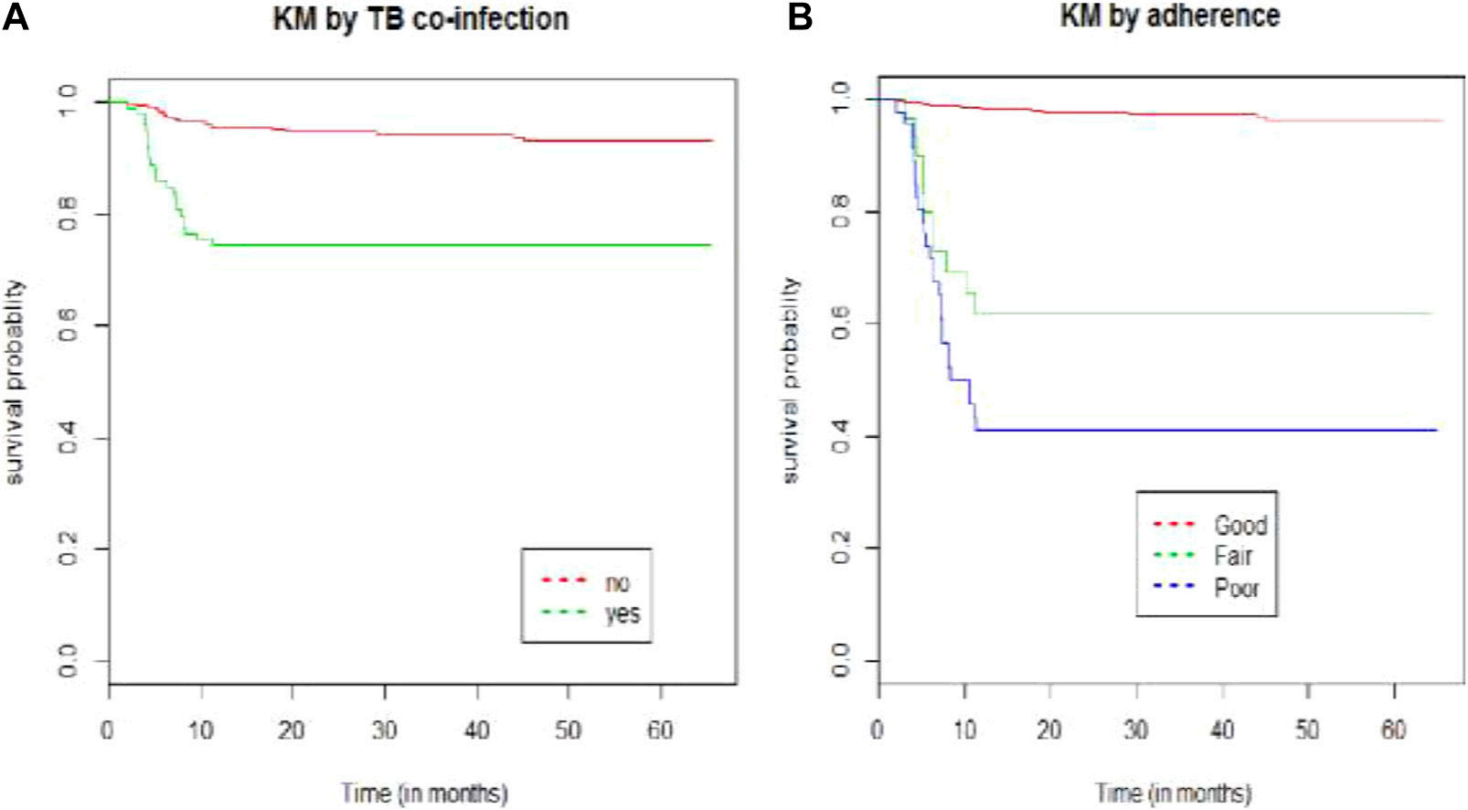
Kaplan–Meier survival curve by **(A)** TB co-infection and **(B)** adherence for HIV/AIDS patients on ART at public hospitals in Eastern Ethiopia from 2013–2019.

The Kaplan–Meier survival estimate revealed that the hazards of developing ART adverse drug reactions were higher among patients who had TB co-infection and patients presented with poor baseline adherence status (Figure 1).

The median follow-up time was 31.3 months [inter-quartile range (IQR), (18.3, 47.5)]. Of the 561 participants, 54 (9.6%) (95% CI: 7.2–12.1%) patients had developed ART adverse drug reactions with 1,514.2 persons-years (PYs) of observations. The incidence density was 3.5/100 PYs with a 95% CI of 2.7, 4.6. About 24 (44.4%), 50 (92.6%), and 52 (96.3%) adverse drug reaction occurred within the first half, 1, and 2 years of the initiation of ART, respectively.

### Bi-Variable and Multivariable Cox Proportional Hazard Analysis

In the bi-variable Cox regression analysis, WHO RVI stage, adherence, BMI, initial ART regimen, comorbidity, TB co-infection, CD4 count, OIP, place of residence, and functional status were significantly associated with the time to ART adverse drug reaction at a *p-*value of 0.2. In the multivariable analysis, the initial ART regimen (TDF, 3TC, and EFV) [AHR = 0.3, 95% CI 0.1–0.7], fair adherence [AHR = 8.8, 95% CI 3.3–23.2], poor adherence [AHR = 7.8, 95% CI 3.1–19.5], moderate BMI at baseline [AHR = 4.4, 95% CI 1.8–11.0], severe BMI [AHR = 2.8, 95% CI 1.1–6.7], WHO stage II [AHR = 3.7, 95% CI 1.2–11.3], and WHO stage IV [AHR = 6.3, 95% CI 2.0–19.8] were significant predictors of the time to ART adverse drug reaction.

For HIV/AIDS patients whose baseline ART regimen was TDF, 3TC, and EFZ, the hazards of developing ART adverse drug reactions were decreased by 70% (AHR = 0.3, 95% CI [0.1–0.7]) at the baseline as compared with patients taking AZT, 3TC, and NVP at baseline while keeping other variables constant.

Adjusting for other variables in the model, the hazard of experiencing ART adverse reactions was 3.7 times [AHR = 3.7, 95% CI (1.2–11.3)] and 6.3 times higher [AHR = 6.3, 95% CI (2.0, 19.8)] for RVI patients with baseline WHO RVI stage II and stage IV, respectively, than those with baseline RVI stage I.

Adjusting for other variables, the hazards of experiencing ART adverse reactions were 4.4 and 2.8 times higher among patients with moderate [AHR = 4.4, 95% CI (1.8–11.0)] and severe [AHR = 2.8, 95%CI (1.1, 6.8)] baseline BMI, respectively, than those with normal BMI at baseline.

Adjusting for other variables, patients with fair and poor baseline adherence status were at 8.8 and 7.8 times higher risk of developing ART adverse reactions [AHR = 8.8, 95% CI (3.3, 23.2) and AHR = 7.8, 95% CI (3.1, 19.5)], respectively, than those patients with good baseline adherence (Table 3).

**TABLE 3:** Predictors of the time to ART ADR among HIV/AIDS patients on ART at public hospitals in Eastern Ethiopia, 2013–2019.

Variables	Category	Event	Censored	CHR (95%CI)	AHR (95%CI)
Residence	Urban	20	331	Ref.	Ref.
	Rural	34	176	2.9 [1.7, 5.2]	1.6 [0.8, 2.9]
ART regimen	AZT, 3TC, NVP	15	15	Ref.	Ref.
	AZT, 3TC, EFV	13	11	1.1 [0.6, 2.4]	0.9 [0.4, 2.2]
	TDF, 3TC, NVP	3	15	0.3 [0.1, 1.0]	0.7 [0.1, 2.9]
	TDF, 3TC, EFV	23	466	0.1 [0.0, 0.1]	**0.3 [0.1, 0.7]**
CD4 count	<200	30	173	Ref.	Ref.
	≥200	24	334	0.4 [0.2, 0.7]	0.8 [0.4, 1.6]
HIV/AIDS stage	Stage I	6	279	Ref.	Ref.
	Stage II	8	84	4.4 [1.5, 12.6]	**3.7 [1.2, 11.3]**
	Stage III	24	134	7.7 [3.1, 18.8]	2.3 [0.8, 6.5]
	Stage IV	16	10	42.5 [16.6, 108.8]	**6.3 [2.0, 19.8]**
BMI	Normal	15	395	Ref.	Ref.
	Moderate	16	66	5.9 [2.9, 11.8]	**4.4 [1.8, 11.0]**
	Severe	23	46	10.8 [5.6, 20.6]	**2.8 [1.1, 6.8]**
TB co-infection	No	28	433	Ref.	Ref.
	Yes	26	74	4.9 [2.9, 8.3]	1.0 [0.5, 2.1]
OIP	No	19	48	Ref.	Ref.
	Yes	35	459	0.2 [0.2, 0.4]	0.8 [0.3, 1.9]
Functional	Working	16	454	Ref.	Ref.
	Ambulatory	24	48	11.7 [6.2, 22.1]	1.8 [0.7, 4.7]
	Bedridden	14	5	38.5 [18.6, 79.8]	2.7 [0.9–7.7]
Comorbidity	No	35	493	Ref.	Ref.
	Yes	19	14	12.0 [6.8, 21.2]	1.0 [0.5, 2.1]
Adherence	Good	13	469	Ref.	Ref.
	Fair	12	19	20.4 [9.3, 44.9]	**8.8 [3.3, 23.2]**
	Poor	29	19	34.3 [17.7, 66.4]	**7.8 [3.1, 19.5]**

HIV: human immunodeficiency syndrome; AIDS: acquired immunodeficiency syndrome; BMI: body mass index; TB: tuberculosis; OIP: opportunistic infection prophylaxis; CHR: crude hazard ratio; AHR: adjusted hazard ratio; AZT: zidovudine; 3 TC: lamivudine; NVP: nevirapine; EFV: efavirenz; TDF: tenefovir. bold values show factors significantly associated with ART adverse drug reactions.

## Discussion

This study assessed the time to ART adverse drug reaction and its predictors among HIV/AIDS patients. A Cox proportional hazard model was applied to identify the predictors of ART adverse drug reactions. Factors observed to have a significant association with the time to ART adverse drug reactions among HIV/AIDS patients were WHO HIV/AIDS stage, BMI, adherence, and initial ART regimen.

The present study revealed that about 9.6% of the participants had ART adverse drug reactions with an incidence density of 3.5 per 100 PY 95% CI [2.7, 4.6]. This result is in line with previous studies conducted in Ethiopia (4.3/100PY) and Nigeria (4.6/100 PY) (Huang et al., 2012; Kindie et al., 2017). However, the finding of the present study was lower than another retrospective study conducted in India (10.11/100 PY) (Shet et al., 2014) and Hosanna, Ethiopia, which reported an incidence density of 4.88 per 100 person-days (Asfaw and Enquselassie, 2018b). The reasons for this discrepancy might be because of the difference in how the adverse drug reaction was measured and the follow-up period. In the later study, patients were followed-up for 2 years whereas in the present study, patients were followed-up for 5.5 years.

In the present study, most (92.6%) ART adverse drug reactions occurred within 1 year of initiation of ART which is supported by a study in Bahir Dar, Ethiopia (Kindie et al., 2017). The possible explanation for this is most HIV/AIDS patients are presented with complications and advanced HIV disease with severe immunodeficiency states which makes adverse drug reactions more likely. Moreover, an intrinsic metabolic intolerance when patients first started ART might also contribute to early ART adverse drug reactions (Shelburne et al., 2002)**.**

In the present study, cumulative probability of survival without developing adverse drug reactions by the end of the follow-up period was 89% [95% CI 86–92%]. This result is supported by a previous study in Ethiopia (Kindie et al., 2017) and this promising survival probability might be because of an early screening program for TB and other associated comorbidities at the start of ART in the study area and most of the patients took opportunistic infection prophylaxis which might contribute to the survival of patients.

Patients with moderate and severe baseline BMI had higher risks of experiencing ART adverse drug reactions than those with a normal BMI at baseline and this is in agreement with a prospective study conducted in Ethiopia (Bezabhe et al., 2015). This might be because of the BMI’s effect on drug metabolism as it contributes to the efficacy of highly active antiretroviral therapy (HAART) (Li et al., 2019)**.** Thus, HIV/AIDS patients with normal BMI are at a lower risk of developing ART adverse drug reactions.

Adherence status is another significant predictor of the time to ART adverse drug reaction. Patients with fair and poor adherence are at higher risk of experiencing ART adverse drug reactions than those with good adherence status, which is supported by a previous study in South Africa (Masenyetse et al., 2015). A possible explanation for this might be that patients who miss their prescribed doses cannot consistently maintain virologic success and are prone to develop ART adverse drug reactions (Bangsberg et al., 2001).

According to our finding, the hazard of experiencing ART adverse drug reactions is higher among HIV/AIDS patients with baseline WHO HIV/AIDS stages II and IV than those with stage I. This result is in line with previous studies (Eluwa et al., 2012; Shet et al., 2014; Weldegebreal et al., 2016; Bhuvana and Hema, 2017; Kindie et al., 2017) which reported that patients with advanced stages of the disease are at a higher risk of ART adverse drug reactions. This might be related to the inability to resist side effects in patients with advanced stages of disease and possible drug interactions since immune-mediated adverse drug reactions are by far more common in HIV/AIDS patients (Peter et al., 2019).

Hazards of developing ART adverse drug reactions for HIV/AIDS patients whose baseline ART regimen was TDF, 3TC, and EFV are lower than those patients taking AZT, 3TC, and NVP at baseline. This finding is in agreement with previous studies in Uganda (van Leth et al., 2004) and Ethiopia (Weldegebreal et al., 2016) and the reason for this might be due to the higher toxicity profile of AZT and NVP-containing regimen (Wester et al., 2010; Masenyetse et al., 2015).

This study is not without limitations. The main drawback of the present study was the absence of important variables which might have an effect on the time to ART adverse drug reaction. Since the present study was based on retrospective data obtained from patient medical records, important variables such as the socio-economic status, behavioral variables (smoking and alcohol drinking status), and clinical variables (viral load and hemoglobin) which are assumed to be associated with ART adverse drug reactions were not obtained in the record.

In conclusion, most ART adverse drug reactions occurred within 1 year of initiation of ART. The initial ART regimen (TDF, 3TC, and EFV), adherence, HIV/AIDS stage, and body mass index were risk factors for the time to ART adverse drug reaction. The incidence of antiretroviral therapy adverse drug reaction was relatively low with early onset. Close monitoring of clients in clinical stage II and above is required and continuous assessment for improving the detection and management of adverse drug reactions is recommended. Patients with poor adherence need to get continuous counseling to improve their adherence status.

## Figures and Tables

**Figure S1:**
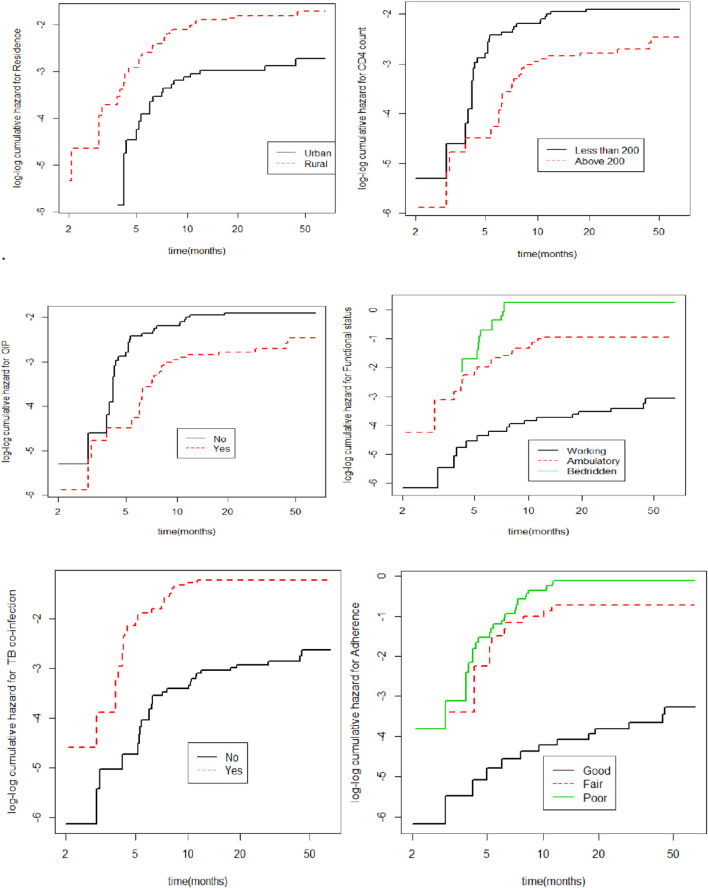
Kaplan-Meier Survival plots for selected baseline covariates.

**Figure S2:**
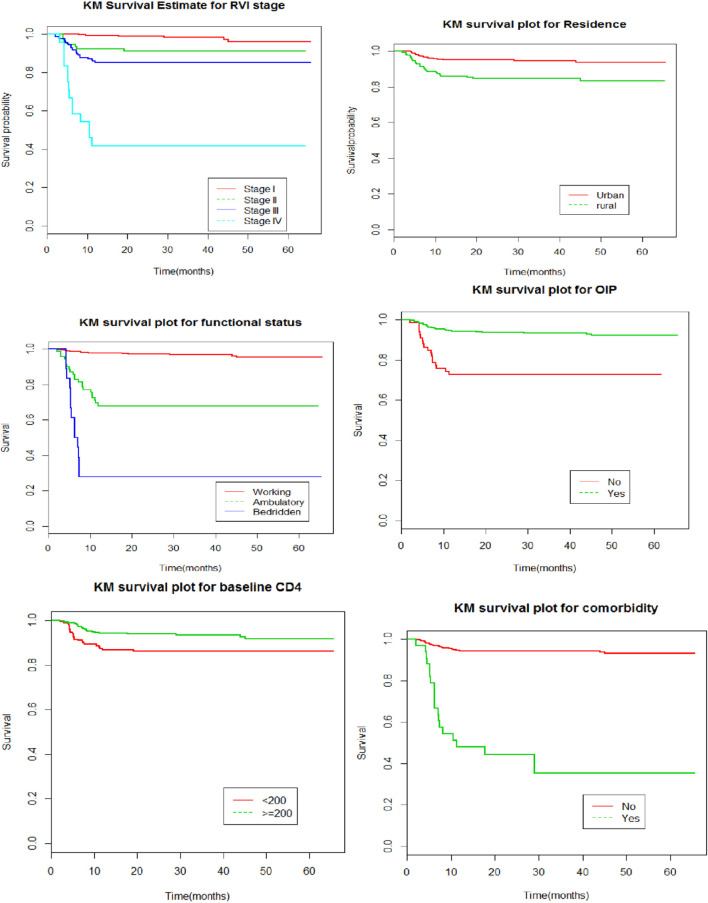
Cumulative hazard plots for checking PHA.

**Figure S3:**
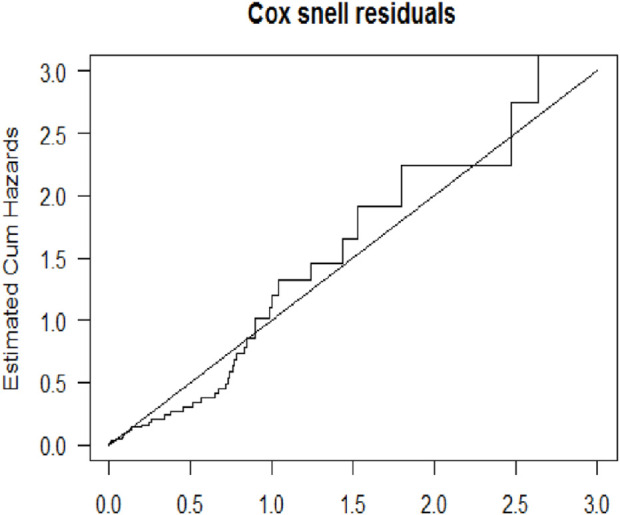
Cox Snell residuals for assessing fitness of Cox proportional hazard model.

## Data Availability

The original contributions presented in the study are included in the article/Supplementary Material; further inquiries can be directed to the corresponding author.

## Abbreviations

AHR, Adjusted Hazard Ratio; AIDS, Acquired Immunodeficiency Syndrome; ART, Antiretroviral Therapy; BMI, Body Mass Index; CHR, Crude Hazard Ratio; CI, Confidence Interval; HAART, Highly Active Antiretroviral Therapy; HIV, Human Immunodeficiency Virus; HR, Hazard Ratio; KM: Kaplan Meier; PHA, Proportional Hazard Assumptions; PY, Person-Year; TB, Tuberculosis; WHO, World Health Organization.
