# Supplementary material for: Time to First Line Antiretroviral Therapy Adverse Drug Reaction and its Predictors Among Adult HIV/AIDS Patients on Treatment in Eastern Ethiopia
Source: Front Pharmacol. 2022 Aug 15;13:922744. doi: 10.3389/fphar.2022.922744 (PMC9421417; doi:10.3389/fphar.2022.922744)
Supplement: Supplementary file 1 [file DataSheet1.docx]

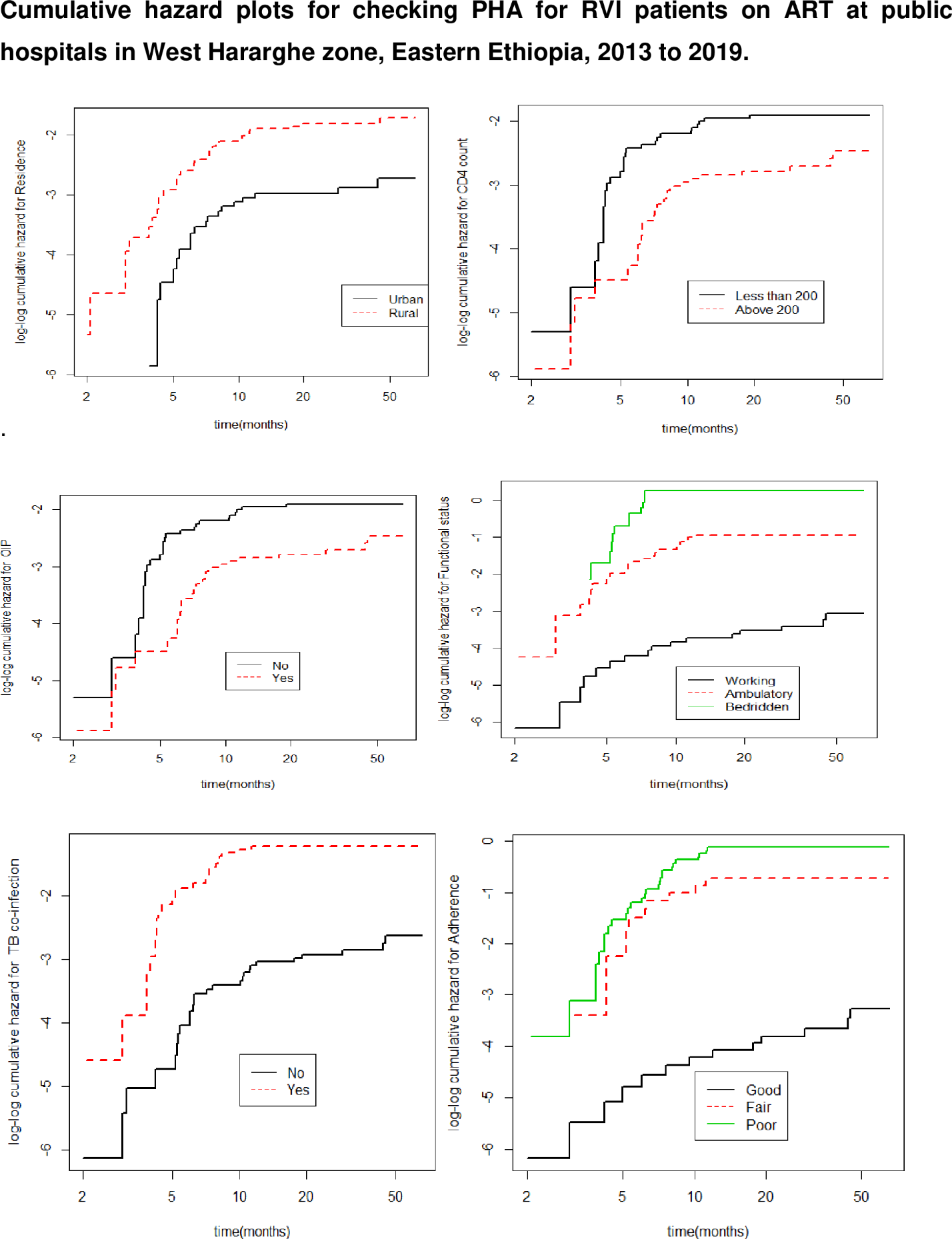
 **S1 Fig. Kaplan-Meier Survival plots for selected baseline covariates.**


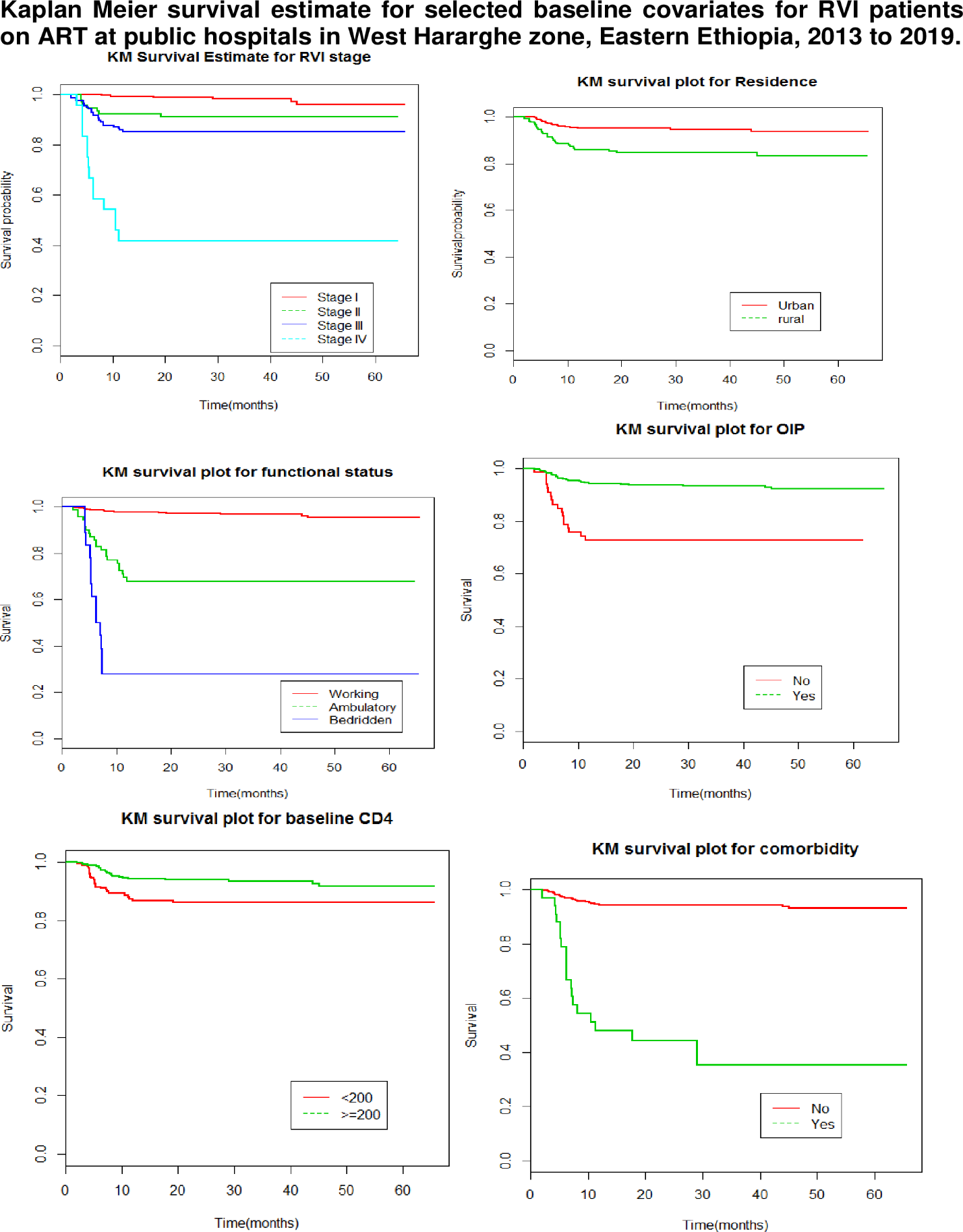
 **S2 Fig. Cumulative hazard plots for checking PHA.**


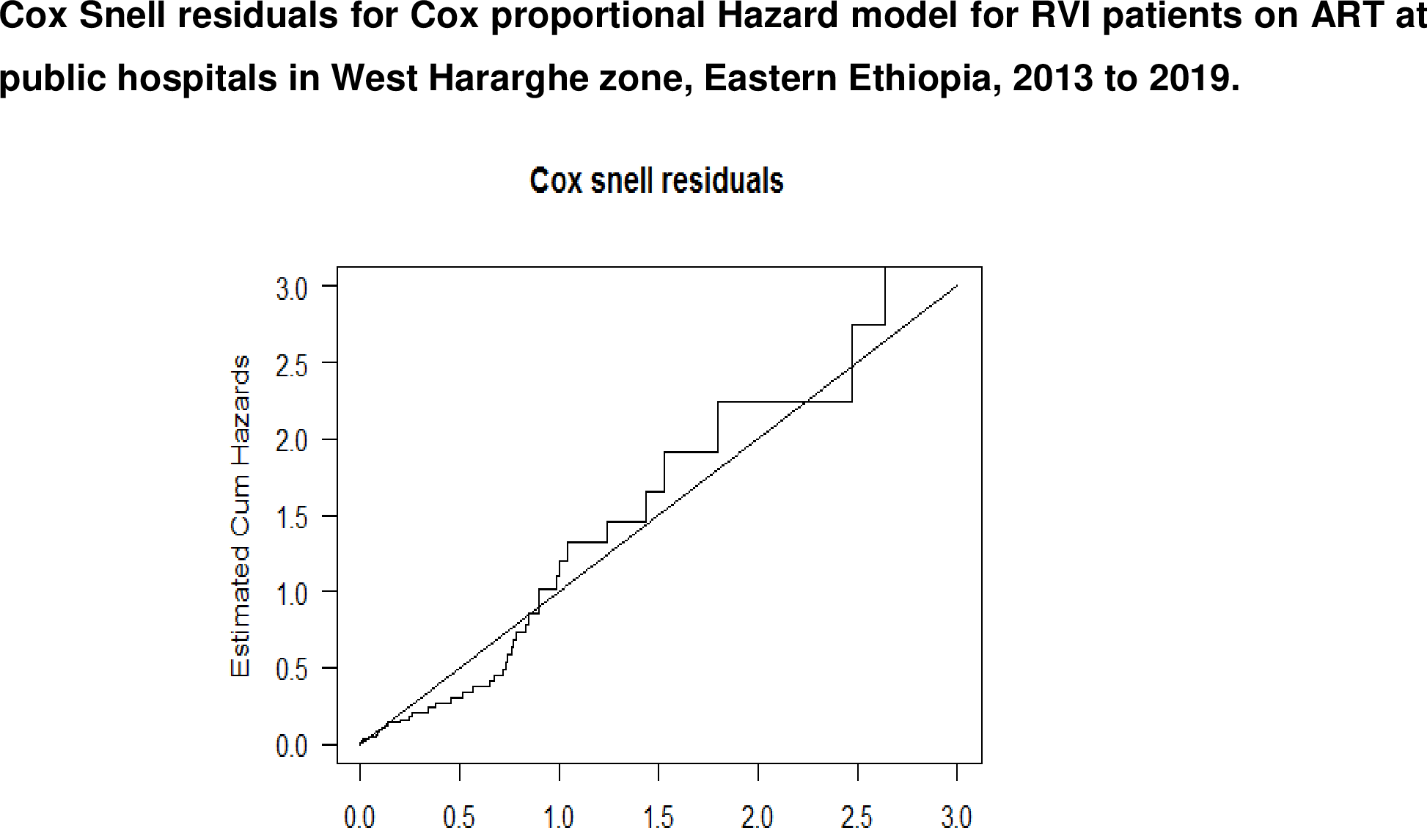


**S3 Fig. Cox Snell residuals for assessing fitness of Cox proportional hazard model.**
